# RETRACTED: Dong et al. Transient Response Characteristics Analysis of High-Power Piezoelectric Transducers. *Micromachines* 2022, *13*, 1638

**DOI:** 10.3390/mi15121438

**Published:** 2024-11-28

**Authors:** Zhaopeng Dong, Liang Xu, Tianyue Yang

**Affiliations:** 1 College of Engineering, Shanghai Ocean University, Shanghai 201306, China; 2 Suzhou Zhitu Technology Co., Ltd., Shanghai 200000, China; 3 Department of Instrument Science and Engineering, Shanghai Jiao Tong University, Shanghai 200240, China

The journal retracts the article titled “Transient Response Characteristics Analysis of High-Power Piezoelectric Transducers” [1], cited above.

Following publication, concerns were brought to the attention of the Editorial Office regarding the accuracy of the authorship list and an overlap between this article [1] and an earlier doctoral thesis [2], produced by a different author. 

Adhering to our standard procedure, the Editorial Office and Editorial Board conducted an investigation. Following communication with the authors, the Editorial Board confirmed that the original authorship list was inaccurate and the presence of a partial overlap with material originally presented within a doctoral thesis [2], without appropriate citation or permission. As a result, the Editorial Board and the authors have decided to retract this paper as per MDPI’s retraction policy (https://www.mdpi.com/ethics#_bookmark30).

This retraction was approved by the Editor-in-Chief of the journal *Micromachines*.

The authors agreed to this retraction.

## References

[1] Dong Z., Xu L., Yang T. (2022). RETRACTED: Transient Response Characteristics Analysis of High-Power Piezoelectric Transducers. Micromachines.

[2] Yang T.-Y. (2020). Basic Research on Driving and Control of High-Power Piezoelectric Ultrasonic Systems. Ph.D. Thesis.

